# Endosymbiosis: Lessons in Conflict Resolution

**DOI:** 10.1371/journal.pbio.0020068

**Published:** 2004-03-16

**Authors:** Jennifer J Wernegreen

## Abstract

Endosymbiotic bacteria live within a host species. There are many and diverse examples of such relationships, the study of which provides important lessons for ecology and evolution

Symbiosis, an interdependent relationship between two species, is an important driver of evolutionary novelty and ecological diversity. Microbial symbionts in particular have been major evolutionary catalysts throughout the 4 billion years of life on earth and have largely shaped the evolution of complex organisms. Endosymbiosis is a specific type of symbiosis in which one—typically microbial—partner lives within its host and represents the most intimate contact between interacting organisms. Mitochondria and chloroplasts, for example, result from endosymbiotic events of lasting significance that extended the range of acceptable habitats for life. The wide distribution of intracellular bacteria across diverse hosts and marine and terrestrial habitats testifies to the continued importance of endosymbiosis in evolution.

Among multicellular organisms, insects as a group form exceptionally diverse associations with microbial associates, including bacteria that live exclusively within host cells and undergo maternal transmission to offspring. These microbes have piqued the interest of evolutionary biologists because they represent a wide spectrum of evolutionary strategies, ranging from obligate mutualism to reproductive parasitism (Buchner 1965; Ishikawa 2003) (Box 1; Table 1). In this issue of *PLoS Biology*, the publication of the full genome sequence of the reproductive parasite Wolbachia allows the first genomic comparisons across this range (Wu et al. 2004).

**Table 1:**
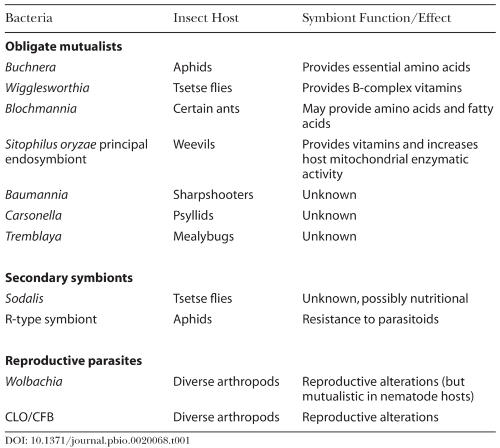
Examples of Bacterial Endosymbionts of Insects

## Lifestyle Extremes in Insect Endosymbionts

**Figure 1:**
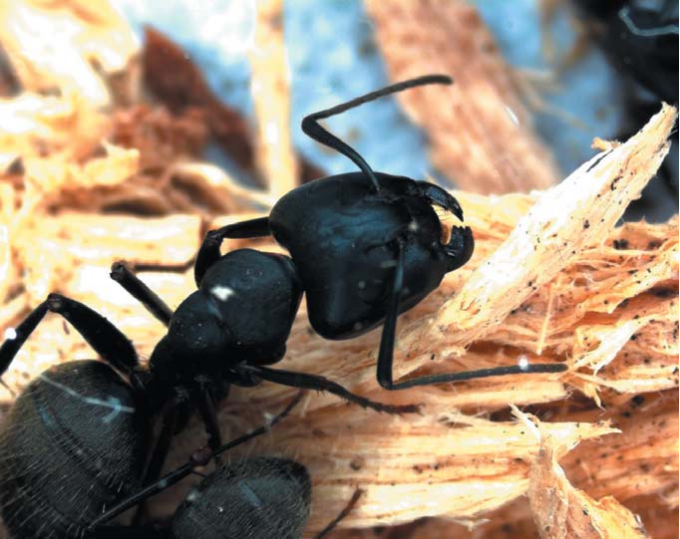
A carpenter ant, Camponotus pennsylvanicus, Hosts the Mutualistic Bacterial Endosymbiont Blochmannia Like all species of the ant genus Camponotus, the wood-nesting C. pennsylvanicus (shown here) possesses an obligate bacterial endosymbiont called Blochmannia. The small genome of Blochmannia retains genes to biosynthesize essential amino acids and other nutrients (Gil et al. 2003), suggesting the bacterium plays a role in ant nutrition. Many Camponotus species are also infected with Wolbachia, an endosymbiont that is widespread across insect groups. (Photo courtesy of Adam B. Lazarus.)

At one end of the spectrum, beneficial endosymbionts provide essential nutrients to about 10%–15% of insects and provide models for highly specialized, long-term mutualistic associations (Figure 1). These ‘primary’ endosymbionts are required for the survival and reproduction of the host, most of which feed on unbalanced diets such as plant sap, blood, or grain, and occur within specialized host cells called bacteriocytes (or mycetocytes) (Baumann et al. 2000; Moran and Baumann 2000). Molecular phylogenetic analyses demonstrate stability of these obligate mutualists over long evolutionary periods, ranging from tens to hundreds of millions of years. By allowing their hosts to exploit otherwise inadequate food sources and habitats, the acquisition of these mutualists can be viewed as a key innovation in the evolution of the host (Moran and Telang 1998). Owing to their long-term, stable transmission from generation to generation (vertical transmission), these cytoplasmic genomes have been viewed as analogs to organelles.

By contrast, reproductive parasites of insects, including Wolbachia (O'Neill et al. 1998) and the more recently discovered endosymbiont in the Bacteroidetes group (also called CFB or CLO) (Hunter et al. 2003), propagate in insect lineages by manipulating host reproduction. These maternally inherited bacteria inflict an impressive arsenal of reproductive alterations to increase the frequency of infected female offspring, often at the expense of their hosts. Such mechanisms include cytoplasmic incompatibility, parthenogenesis, and male killing or feminization. As parasites, these bacteria rely on occasional horizontal transmission to infect new populations (Noda et al. 2001) and, by directly altering reproductive patterns, may be a causative agent of host speciation (Bordenstein et al. 2001).

Comparative molecular analysis of insect endosymbionts over the past decade has provided new insights into their distribution across hosts, their varying degrees of stability within host lineages (ranging from cospeciation to frequent host-switching), and their impressive genetic diversity that spans several major bacterial groups. More recently, studies in genomics of obligate mutualists—and now Wolbachia—illuminate mechanisms of host–symbiont integration and the distinct consequences of this integration in various symbiotic systems. In addition, since hosts and symbionts often have different evolutionary interests, the diverse features of insect–bacterial associations can be understood as different outcomes in the negotiation of genetic conflicts. Some recent highlights and tantalizing research areas are described below.

## Endosymbiont Genomes: Spanning the Gamut from Static to Plastic

The distinct lifestyle of endosymbionts has clear effects on rates and patterns of molecular evolution. Compared to free-living relatives, endosymbionts are thought to have reduced effective population sizes due to population bottlenecks upon transmission to host offspring and, in the case of obligate mutualists that coevolve with their hosts, limited opportunities for gene exchange. The nearly neutral theory of evolution (Ohta 1973) predicts accelerated fixation of deleterious mutations through random genetic drift in small populations, a phenomenon that has been observed in endosymbionts (Moran 1996; Lambert and Moran 1998). Over time, this lifestyle-associated accumulation of deleterious mutations may negatively affect the fitness of both the host and symbiont.

It is increasingly clear the distinct lifestyle of endosymbionts also shapes the architecture and content of their genomes, which include the smallest, most AT-rich bacterial genomes yet characterized (Andersson and Kurland 1998; Moran 2002). A common theme is substantial gene loss, or genome streamlining, which is thought to reflect an underlying deletion bias in bacterial genomes combined with reduced strength or efficacy of selection to maintain genes in the host cellular environment. As a result of gene loss, these bacteria completely rely on the host cell for survival. Because they cannot be easily cultured apart outside of the host for traditional genetic or physiological techniques, analysis of genome sequence offers a valuable tool to infer metabolic functions that endosymbionts have retained and lost and to elucidate the steps in the evolutionary processes of genome reduction.

Since 2000, full genome sequences have been published for Buchnera of three aphid host species, Wigglesworthia of tsetse flies, and Blochmannia of ants (Shigenobu et al. 2000; Akman et al. 2002; Tamas et al. 2002; Gil et al. 2003; van Ham et al. 2003). Parallels among these mutualist genomes include their small size (each smaller than 810 kb), yet retention of specific biosynthetic pathways for nutrients required by the host (for example, amino acids or vitamins). However, genomes also show signs of deleterious deletions. Early gene loss in Buchnera involved a few deletions of large contiguous regions of the ancestral genome and often included genes of unrelated functions (Moran and Mira 2001). These ‘large steps’ imply that genome reduction involved some random chance (due to the location of genes in the ancestral chromosome) and selection acting on the combined fitness of large sets of genes, rather than the fitness of individual loci. Such deletions are apparently irreversible in obligate mutualists, which lack recombination functions and genetic elements, such as prophages, transposons, and repetitive DNA that typically mediate gene acquisition. The scarcity of these functions, combined with limited opportunities to recombine with genetically distinct bacteria, may explain the unprecedented genome stability found in Buchnera compared to all other fully sequenced bacteria (Tamas et al. 2002) and a lack of evidence for gene transfer in other mutualist genomes. Stability also extends to the level of gene expression, as obligate mutualists have lost most regulatory functions and have reduced abilities to respond to environmental stimuli (Wilcox et al. 2003).

The Wolbachia genome presented in this issue allows the first genome comparisons among bacteria that have adopted divergent evolutionary strategies in their associations with insects (Wu et al. 2004). Like other parasites, but unlike long-term mutualists, Wolbachia may experience strong selection for phenotypic variation, for example, to counter improved host defenses, to compete with distinct Wolbachia strains that coinfect the same host, or to increase its transmission to new host backgrounds. High levels of recombination in Wolbachia (for example, Jiggins et al. 2001) may allow rapid genetic changes in this parasite and may be catalyzed by the exceptionally high levels of repetitive DNA and mobile elements in its genome (Wu et al. 2004). Other bacteria that colonize specialized niches for long periods and lack co-colonizing strains also possess high levels of repetitive chromosomal sequences. For example, among ulcer-causing Helicobacter pylori in primate guts, repetitive DNA mediates intragenomic recombination and may provide an important source of genetic variation for adaptation to dynamic environmental stresses (Aras et al. 2003). The potential contributions of repetitive DNA and phage to intragenomic and intergenomic recombination in Wolbachia are exciting areas of research (Masui et al. 2000). The Wolbachia genome also provides a valuable tool for future research to test whether plasticity extends to gene content variation among Wolbachia strains and labile gene expression patterns.

Between these two extremes of obligate mutualism and reproductive parasitism lies a spectrum of secondary symbionts of insects, most of which have not yet been studied in detail. Such ‘guest’ microbes transfer among diverse host species (Sandström et al. 2001), may provide more subtle or occasional benefits (for example, relating to host defense against parasitoids [Oliver et al. 2003]), and could represent an intermediate stage between a free-living lifestyle and obligate endosymbiosis. Genome-level data from these secondary symbionts promise to shed light on the range of lifestyles between obligate mutualism and reproductive parasitism and on the early stages in the transition to each. Microarray-based comparisons of gene content among Escherichia coli, a facultative mutualist of tsetse flies (Sodalis glossinidius), and a relatively young mutualist of weevils (Sitophilus oryzae primary endosymbiont [SOPE]) show that genome streamlining in the endosymbionts may preclude extracellular existence, and highlight modifications in metabolic pathways to complement specific host physiology and ecology (Rio et al. 2003). In addition, these endosymbionts may employ similar mechanisms as intracellular parasites in overcoming the shared challenges of entering host cells, avoiding or counteracting host defense mechanisms, and multiplying within a host cellular environment (Hentschel et al. 2000). The rapidly growing molecular datasets for secondary (or young primary) insect endosymbionts have identified pathways that are considered to be required for pathogenicity, such as Type III secretion (Dale et al. 2001, 2002). Such pathways may therefore have general utility for bacteria associated with host cells and may have evolved in the context of beneficial interactions.

## Genetic Conflicts and Host–Symbiont Dynamics

Given their diverse evolutionary strategies, insect endosymbionts also provide a rich playing field to explore genetic conflicts (Frank 1996a, 1996b), which might involve the mode of symbiont transmission, the number of symbionts transmitted, and the sex of host offspring. Genetic conflicts described between organelle and nuclear genomes of the same organism (Hurst 1995) can provide a context to understand the evolutionary dynamics of insect–bacterial associations and the diverse outcomes of these relationships. For example, the uniparental (maternal) mode of inheritance of both mitochondria and insect endosymbionts may reflect host defense against invasion by foreign microbes with strong deleterious effects, which spread more easily under biparental inheritance (Law and Hutson 1992).

Host–symbiont conflicts over offspring sex ratio are quite apparent in reproductive parasites (Vala et al. 2003). While the bacteria favor more female offspring and employ a variety of mechanisms to achieve this, the host typically favors a more balanced sex ratio. This conflict may lead to changes in the host that counter the symbiont's effect on sex ratio. For example, the spread of Wolbachia in a spider mite population caused selection on host nuclear genes that decrease the symbiont-induced sex ratio bias (Noda et al. 2001).

Obligate mutualists also experience genetic conflicts with the host regarding transmission mode and number. In general, symbionts generally favor dispersal out of the host to avoid competition with their close relatives, while hosts are expected to restrict symbiont migration and thus reduce the virulent tendencies (Frank 1996b). In obligate mutualisms, there may be little room for negotiation. For example, the highly conserved, host-controlled determination of aphid bacteriocytes (Braendle et al. 2003) and the phylogenetic congruence observed in numerous studies suggest that aphids have won this conflict over symbiont transfer. However, the number of bacteria transmitted may be more flexible and is known to vary among aphid taxa (Mira and Moran 2002). Models indicate that the fixation rate for symbiont-beneficial (selfish) mutations increase with the number of symbionts transmitted, reflecting greater efficacy of selection among bacteria within a given host (Rispe and Moran 2000).

## Prospects

In sum, the past few years have witnessed a surge of new empirical and theoretical approaches to understand the dynamics of bacterial–insect relationships. These tools have shed light on the roles of recombination, selection, and mutation on endosymbiont genome evolution and have highlighted parameters that shape the outcome of genetic conflicts between hosts and symbionts. These data provide a foundation for studying the evolution of mutualism and parasitism and modes of transitions between them. In the near future, we can look forward to full genome sequences that span a broader ecological and phylogenetic diversity of endosymbionts and provide a richer comparative framework to test existing models and develop new ones.

Developments in endosymbiosis are important not only to questions in basic research, but may have important practical applications. Blood-feeding insects such as mosquitoes and tsetse flies are vectors for parasites that cause significant global infectious diseases such as malaria, dengue virus, and trypanosomiasis, many of which have frustrated attempts at vaccine development. The same insects that transmit these devastating human parasites often possess a diversity of mutualistic and parasitic bacterial endosymbionts. A very promising and urgent area of endosymbiont research is the manipulation of these bacteria to control vector populations in the field. Current studies already provide evidence that endosymbiont manipulation is a promising strategy to reduce the lifespan of the insect vector or limit its transmission of disease-causing parasites (Aksoy et al. 2001; Brownstein et al. 2003). Each advance in our understanding of endosymbiont genomics and evolutionary dynamics represents one step closer to that goal.

##

**Box 1. Glossary**

**Endosymbiont:**A symbiont that lives inside of its host, often within host cells (intracellular symbiont).

**Facultative mutualist:** A beneficial symbiont that associates with the host, but can also live apart from it. Examples include Rhizobium spp. that associate with legumes, but also have a free-living stage to their life cycle.

**Obligate mutualist:** A beneficial symbiont that lives exclusively with its host and depends on the host for survival. Examples include many nutritional endosymbionts of insects, which cannot survive outside of the insect host cell. These associations are reciprocally obligate when the host cannot survive without the endosymbiont.

**Parasite:** A symbiont that has a negative effect on host fitness, in contrast to a mutualist, which increases host fitness.

**Reproductive parasite:** A symbiont that manipulates host reproduction to its own benefit, but at the expense of host fitness. Reproductive parasites typically bias offspring toward infected females.

**Symbiosis:** An association between two more species.

## Abbreviation

SOPE: Sitophilus oryzae primary endo-symbiont
